# The ‘Narcissus Effect’: Top-down alpha-beta band modulation of face-related brain areas during self-face processing

**DOI:** 10.1016/j.neuroimage.2020.116754

**Published:** 2020-06

**Authors:** Elisabet Alzueta, María Melcón, Ole Jensen, Almudena Capilla

**Affiliations:** a Departamento de Psicología Biológica y de la Salud, Facultad de Psicología, Universidad Autónoma de Madrid, Madrid, Spain; b Centre for Human Brain Health, School of Psychology, University of Birmingham, Birmingham, United Kingdom

**Keywords:** Self-processing, Self-face, Fusiform gyrus, Attention, Alpha-beta band

## Abstract

Self-related information, such as one’s own face, is prioritized by our cognitive system. Whilst recent theoretical developments suggest that this is achieved by an interplay between bottom-up and top-down attentional mechanisms, their underlying neural dynamics are still poorly understood. Furthermore, it is still matter of discussion as to whether these attentional mechanisms are truly self-specific or instead driven by face familiarity. To address these questions, we used EEG to record the brain activity of twenty-five healthy participants whilst identifying their own face, a friend’s face and a stranger’s face. Time-frequency analysis revealed a greater sustained power decrease in the alpha and beta frequency bands for the self-face, which emerged at late latencies and was maintained even when the face was no longer present. Critically, source analysis showed that this activity was generated in key brain regions for self-face recognition, such as the fusiform gyrus. As in the Myth of Narcissus, our results indicate that one’s own face might have the potential to hijack attention. We suggest that this effect is specific to the self and driven by a top-down attentional control mechanism, which might facilitate further processing of personally relevant events.

## Highlights

• Self-face processing induces a sustained alpha-beta power suppression.
• Alpha power reduction is generated around face-sensitive regions.
• Modulation of the alpha band indicates increased attention to one’s own face.
• Fostered attention is specific to the self and cannot be explained by familiarity.

## 1 Introduction

For centuries, the self has been of great interest for various fields of research, an interest that can most likely be traced back to ancient Greek culture (Morris, 1994). Over the past few years, research on the neural correlates of the self has increased considerably, leading to the development of new theoretical frameworks (Sui and Gu, 2017). This has been partly due to the potential relevance of this issue for health, since recent evidence suggests that self-processing is altered in many neuropsychological (Sui et al., 2015; Sui et al., 2013) and psychiatric disorders (Grimm et al., 2009; Lemogne et al., 2009; Liemburg et al., 2012). This is, for example, evident in the case of depression, in which rumination has been linked to maladaptive self-focused attention (Northoff, 2007; Watkins and Teasdale, 2004)

It is well known that self-related information, such as the self-name (Imafuku et al., 2014; Moray, 1959) or the self-face (Keyes et al., 2010; Keyes and Dlugokencka, 2014; Sui and Humphreys, 2013), is prioritized by our cognitive system (Sui and Rotshtein, 2019). This ‘self-bias’ has been attributed to specific attentional mechanisms that operate during self-processing (Humphreys and Sui, 2016; Kuang, 2016; Sui and Gu, 2017). In fact, self-bias increases (hyper-) and decreases (-hypo) can be observed in patients with damage to brain regions supporting top-down and bottom-up attentional control. On the one hand, lesions in executive control areas, such as the superior temporal and left prefrontal cortices, lead to a ‘hyper-self-bias’. On the other hand, damage to critical areas for self-face processing, such as the hippocampus or the fusiform gyrus, is linked to a ‘hypo-self-bias’, that is, reduced self-prioritization (Sui et al., 2015).

During self-face processing, this self-bias is manifest in the capture (Brédart et al., 2006) and retention of attention (Devue et al., 2009; Wójcik et al., 2018). This effect might reflect the involvement of two different but complementary attentional mechanisms. We propose that an early bottom-up mechanism might explain the attention-capturing properties of the self-face, whereas the later modulation of attention via top-down control might explain the difficulties in disengaging attentional resources from it. Recent research has already found evidence for an early bottom-up attentional capture by the self-face (Alzueta et al., 2019; Bola et al., 2020; Devue and Brédart, 2008; Keyes and Dlugokencka, 2014; Wójcik et al., 2019). However, it is unclear as to whether a complementary top-down attentional mechanism comes into play later. Here, we propose that endogenous attentional resources are indeed specifically allocated to the self-face, hijacking the perceiver’s attention as a result.

Our proposal is in line with the theoretical framework described by Humphreys and Sui (2016), referred to as the Self-Attention Network, as well as with the more recent neural model of the self (Sui and Gu, 2017; see also Ocampo and Kahan, 2016; Vallesi, 2016 for a discussion about the Self-Attention Network). According to this approach, brain regions sensitive to self-related stimuli interact with bottom-up and top-down attentional control networks to orient our attention and shape behaviour. However, some studies have brought into question the notion that this attentional benefit is specific to the self, since similar effects have also been observed in response to familiar faces (Bortolon et al., 2018; Devue et al., 2009). Under these circumstances, attentional engagement could simply be driven by the high degree of familiarity of one’s own face, and not by the operation of the Self-Attention Network, calling into question the current neural model of the self (Humphreys and Sui, 2016; Sui and Gu, 2017). In the present study, we aimed to discern whether self-bias is truly specific to the self or rather driven by familiarity, thus contributing towards the validation of the neural model of the self.

Importantly, evidence supporting the Self-Attention Network derives mainly from functional Magnetic Resonance Imaging (fMRI) studies. This imposes some limitations in characterizing the temporal dynamics of the attentional mechanisms underlying self-face processing. Hence, in the present study we used electroencephalography (EEG) to chart the temporal dynamics of oscillatory brain activity with a higher resolution. We hypothesize a key role of alpha (8–13 ​Hz) and gamma band (>30 ​Hz) rhythms, given their involvement in top-down controlled gating (Bonnefond and Jensen, 2015; Jensen et al., 2014; Klimesch, 2012). Modulation of alpha band power has consistently been found to be an index of attentional deployment (Gould et al., 2011; Thut et al., 2006; Worden et al., 2000). Moreover, alpha power suppression in specialized sensory regions, such as the extrastriate, visual, auditory or somatosensory cortices, facilitates the perception of visual (Capilla et al., 2014), auditory (Müller and Weisz, 2012) and tactile (Haegens et al., 2011) stimuli, respectively. This attentional bias might reflect an increase in the excitability levels of the brain regions specialized in processing incoming events (Capilla et al., 2014), resulting in an increase in gamma band power (Fries et al., 2007; Fries et al., 2001; Jokisch and Jensen, 2007).

Therefore, the purpose of the present study was to investigate the oscillatory mechanisms underlying self-face processing. To this end, participants performed a facial recognition task while their brain activity was recorded using EEG. In addition to the participant’s own face, we employed two facial stimuli as controls with varying degrees of familiarity (i.e. a friend’s face and an unknown face). We hypothesize that, unlike other faces, the self-face will induce sustained attentional engagement, as indexed by a decrease in alpha and an increase in gamma band power in the specialized brain regions dedicated to face processing.

## 2 Materials and methods

### 2.1 Participants

Thirty healthy volunteers (22.7 ​± ​3.6 years old, mean ​± ​SD; 12 males) with normal or corrected-to normal vision participated in the study. All participants were right-handed according to the Edinburgh Handedness Inventory (Oldfield, 1971) and provided informed written consent. Five participants were not included in the analyses due to low quality EEG recordings. Thus, the remaining sample was composed of twenty-five participants (22.7 ​± ​3.8 years old; 9 males). The study was approved by the Ethics Committee of the Autonoma University of Madrid, and conducted in compliance with the declaration of Helsinki.

### 2.2 Stimuli

**Fig. 1:**
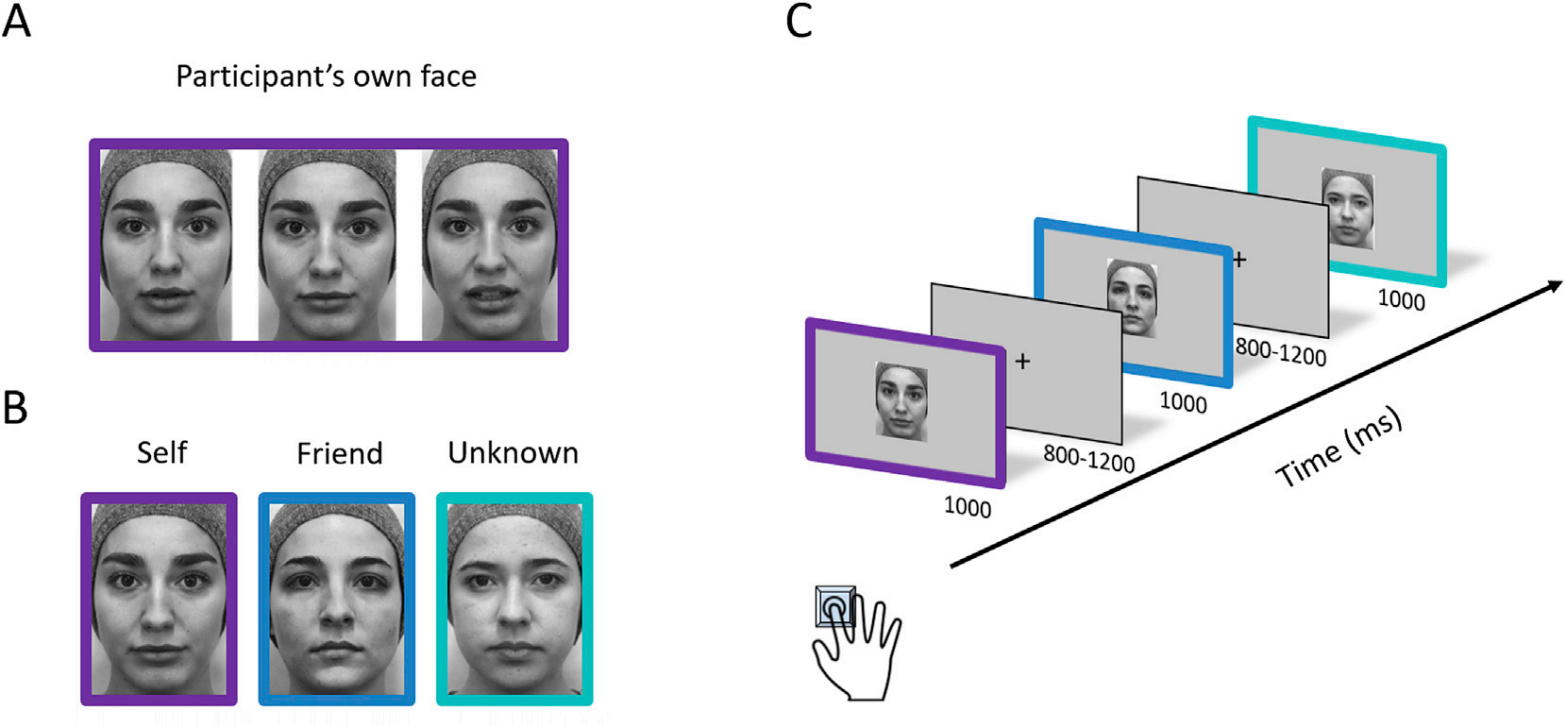
**Experimental stimuli and procedure.** (A) Examples of different stimulus variants for one face: the left-side image shows a neutral expression; the other two images show the same person articulating speech sounds. (B) Examples of face stimuli employed in each experimental condition: Self, Friend and Unknown face. (C) Sequential presentation of face stimuli during the task. Note: coloured frames are shown for illustrative purposes only; they were not presented during the experiment.

The experimental procedure and stimuli have been described in detail in Alzueta et al. (2019). The stimuli were faces of the same gender with three levels of familiarity: (1) ‘Self’, i.e. one’s own face, (2) ‘Friend’, i.e. that of a classmate with whom the participant has regular contact and has known for at least one year (see, for example, Keyes et al., 2010), and (3) ‘Unknown’, i.e. a stranger’s face. At the end of the experimental session, we asked each participant to confirm that the stranger’s face was actually unknown to them. Participants were photographed (Canon EOS 500D) under studio lighting (Neewer®). To enhance stimulus variability, 15 different photographs were taken for each participant, maintaining a neutral expression and articulating several speech sounds (Fig. 1A). In addition, participants were photographed wearing a grey woollen hat to naturally remove external facial features (Fig. 1B). In order to control for differences between stimuli across conditions each participant’s face belonged to each condition once: as self-face (in mirror-reversed orientation; Brédart, 2003) in his/her own experiment, and as a friend’s and stranger’s face in other participants’ experiments. Each photograph was edited using Adobe Photoshop® in three steps. First, the images were centred by converging an imaginary horizontal line through the pupils and the vertical bisection of the face across all images. Second, all images were cropped at 247 x 350 pixels. Third, they were converted to grayscale. Finally, we used the SHINE toolbox (Willenbockel et al., 2010) running under Matlab 2015b to control for low-level image properties, such as luminance, contrast and spatial frequency. Some examples of the experimental stimuli can be seen in Fig. 1.

### 2.3 Experimental procedure

Participants performed the experiment in a dimly-lit, silent, and spacious room. The experimental task was run using Psychtoolbox (Brainard, 1997), and presented on a computer screen located at a viewing distance of 50 ​cm. Previously processed face stimuli were randomly presented for 1000 ​ms, subtending a visual angle of 6.8° x 9.4°. During the inter-stimulus interval, a fixation point on a grey screen was displayed. The length of these intervals ranged randomly between 800 and 1200 ​ms (see Fig. 1C).

The task consisted of identifying the images as one’s own face, a friend’s face or a stranger’s face. Participants provided their response by pressing a key on a numerical keyboard with either the index, middle, or ring finger. The correspondence between key and condition was randomly assigned across participants. Each participant completed a total of 450 trials (15 images x 10 repetitions x 3 conditions), lasting approximately 17 ​min. The experiment was administered in 3-min blocks with short breaks between them to avoid fatigue. Participants were given verbal instructions to avoid eye blinking and invited to remain still and relaxed during the breaks. In order to familiarize the participants with the task and the response key, they completed a 24-trial practice session prior to the experiment. In this session, the stimuli consisted of a model face in which internal features were blurred and replaced by a label indicating identity (‘me’, ‘friend’ or ‘stranger’).

### 2.4 Statistical analysis of behavioural data

The effect of face identity (Self, Friend, Unknown) on behavioural responses (hits and response times) was tested by means of one-way repeated measures analysis of variance (ANOVA). Greenhouse-Geisser correction for non-sphericity was applied when required. We subsequently conducted post-hoc pairwise comparisons to detect differences between conditions. Effect sizes were estimated using the partial eta-square (*η*^2^_p_) method. These analyses were carried out with SPSS 15.0.

### 2.5 EEG recording

The EEG signal was acquired using BioSemi bioactive electrode caps with 128 EEG channels. Four additional electrodes were employed to register the horizontal and vertical EOG. Active electrode offsets were kept below 25–30 ​mV. The data were low-pass filtered online at 100 ​Hz and digitized at a sampling rate of 512 ​Hz.

### 2.6 EEG data analysis

#### 2.6.1 Preprocessing

EEG data analysis was conducted using the Fieldtrip toolbox (Oostenveld et al., 2011) and in-house Matlab code. We first re-referenced the EEG signal to the common average. The data were then segmented into 3000 ​ms long epochs, starting 1000 ​ms before stimulus onset. These long epochs were used with the aim of avoiding edge effects in the time-frequency analysis. Only the trials on which the participants responded correctly were subjected to further analysis.

Artefact rejection was carried out in three steps. Firstly, the EEG data were visually inspected trial-by-trial. Trials contaminated with artefacts such as cable movement, swallowing, or muscular activity were manually discarded. Trials containing blinks or eye-movements during stimulus presentation were also rejected as they may affect visual processing. This procedure resulted in an approximately equal number of trials per condition (123.5 ​± ​15.3 trials in the Self-face condition, 121.0 ​± ​15.7 trials in the Friend condition, and 120.8 ​± ​16.3 trials in the Unknown condition). Secondly, remaining ocular artefacts were reduced in the EEG signal using Independent Component Analysis (‘runica’ algorithm implemented in Fieldtrip). Finally, noisy channels were interpolated using the signal recorded by neighbouring electrodes.

#### 2.6.2 Time-frequency analysis of power

Time-frequency representations of power were calculated for each trial using a (multi-) taper approach with sliding time windows (Percival and Walden, 1993). We analysed lower and higher frequency bands separately to optimize temporal and spectral resolution. For lower frequencies (2.5–30 ​Hz), we employed a moving window of 400 ​ms in 50 ​ms steps and a Hanning taper, leading to ±2 ​Hz spectral smoothing. For higher frequencies (30–100 ​Hz), we made use of the multitaper approach (using discrete prolate spheroidal sequences). We applied a 200 ​ms sliding window, in 50 ​ms time steps, resulting in ±10 ​Hz smoothing. Subsequently, time-frequency maps were averaged across trials for each condition and normalized by calculating the relative change from baseline (from 500 to 200 ​ms before stimulus onset).

#### 2.6.3 Cluster-based statistics for time-frequency power

Statistical analysis of the time-frequency maps was conducted using non-parametric cluster-based permutation tests (Maris and Oostenveld, 2007) to control for multiple comparisons. We first tested for differences between the three conditions using a cluster-based F-test, and subsequently identified specific differences between pairs of conditions by means of cluster-based permutation t-tests. The cluster-based F-test was conducted on the three-dimensional data, i.e. all (channel-frequency-time)-triplets, to identify differences between the three conditions. We then reduced the data to two-dimensions, i.e. (channel-time)-pairs over each frequency range of interest, and conducted pairwise cluster-based t-tests to isolate specific differences between conditions.

The procedure for the cluster-based F/*t*-test analyses was as follows. Adjacent electrodes, (frequency-bins for the F-test) and time-points with p-values below 0.05 were grouped into clusters. Cluster-based statistics were computed as the sum of F/t-values within a cluster. We then determined the significance probability of the cluster statistic by means of a permutation test. The permutation distribution was created by randomly splitting the data set into two subsets and extracting the maximum cluster-level statistic. We repeated this procedure 1000 times to obtain a reference distribution of test statistics. The cluster p-value was then obtained as the proportion of permutations above the observed cluster-based statistic.

#### 2.6.4 Source analysis

The final step of the analysis aimed to localize the brain sources underlying significant cluster-level time-frequency effects. We applied beamforming (Gross et al., 2001; Van Veen, Van Drongelen, Yuchtman and Suzuki, 1997) to estimate the oscillatory activity in the standard MNI brain (see Capilla et al., 2016; Capilla et al., 2014 for details). We employed a standard boundary element method (BEM) volume conduction model (Oostenveld et al., 2003), as well as standard 10–05 electrode positions. The standard MRI was segmented into 10 ​mm voxels, and we computed the lead fields for each of them.

The EEG signal was band-pass filtered at the frequency of interest (i.e. 8-13 ​Hz for alpha, and 13–30 ​Hz for beta). We then extracted data segments corresponding to the extension of the statistically significant cluster (1.2–1.6 ​s for alpha, and 0.7–1.3 ​s for beta), as well as 200 ​ms segments from baseline for subsequent normalization. These segments were concatenated to calculate the single-trial covariance matrix. This was used to compute the spatial filter coefficients using linearly constrained minimum variance (LCMV) beamformer (Van Veen et al., 1997). Regularization (lambda) was set to 10%.

Subsequently, we projected the sensor-level band-pass filtered signal of each trial into each voxel of source-space by means of the spatial filter corresponding to a dipole with fixed optimal orientation. We then computed the amplitude envelope for each trial (i.e. the absolute value of the Hilbert transform), and averaged these across trials and time for each condition separately. To control for the centre of the head bias, source-level activity was normalized as relative change from baseline for each voxel. Finally, brain activation volumes were averaged across participants in order to identify voxels showing spatial maxima/minima.

#### 2.6.5 Relation between alpha band power and behavioural performance

Finally, we tested whether the attentional capture induced by the self-face (as indexed by alpha band suppression) could have an influence on behavioural performance on the subsequent trial. We focused our analysis on the alpha band as this showed a longer-lasting effect. Using an analysis strategy similar to that described by Capilla et al. (2014), we first selected the voxel in source-space with the minimum value of alpha band power for all conditions. Then, for each subject, we computed the single-trial alpha band power in the interest time window (i.e. 1.2–1.6 ​s), which was then normalized in terms of relative change with respect to baseline (0.4–0.2 ​s). Given that alpha power is somewhat heterogeneous across individuals, we organized the single trials into quartile bins instead of using alpha power as a continuous variable. This allowed us to avoid possible bias in the analysis due to participants having very high/low levels of alpha power. Finally, we calculated the change in response time with regard to each participant’s mean response time for each quartile bin. Statistical analysis was carried out by means of linear regression across subjects, and multiple comparisons were controlled by a permutation test. In brief, trials were randomly assigned to each quartile bin, and response times computed for each of these. We repeated this procedure 1000 times. In each repetition, the maximum R^2^ value was stored. The resultant distribution of R^2^ values was employed to derive corrected P-values.

## 3 Results

This study aimed to investigate the neural oscillatory dynamics underlying self-face processing. Participants were asked to discriminate between different identities (*Self, Friend, Unknown*) while their brain activity was simultaneously recorded with EEG. We subsequently conducted time-frequency as well as source analysis to identify the attentional mechanisms specific to the self.

### 3.1 Behavioural results

**Fig. 2:**
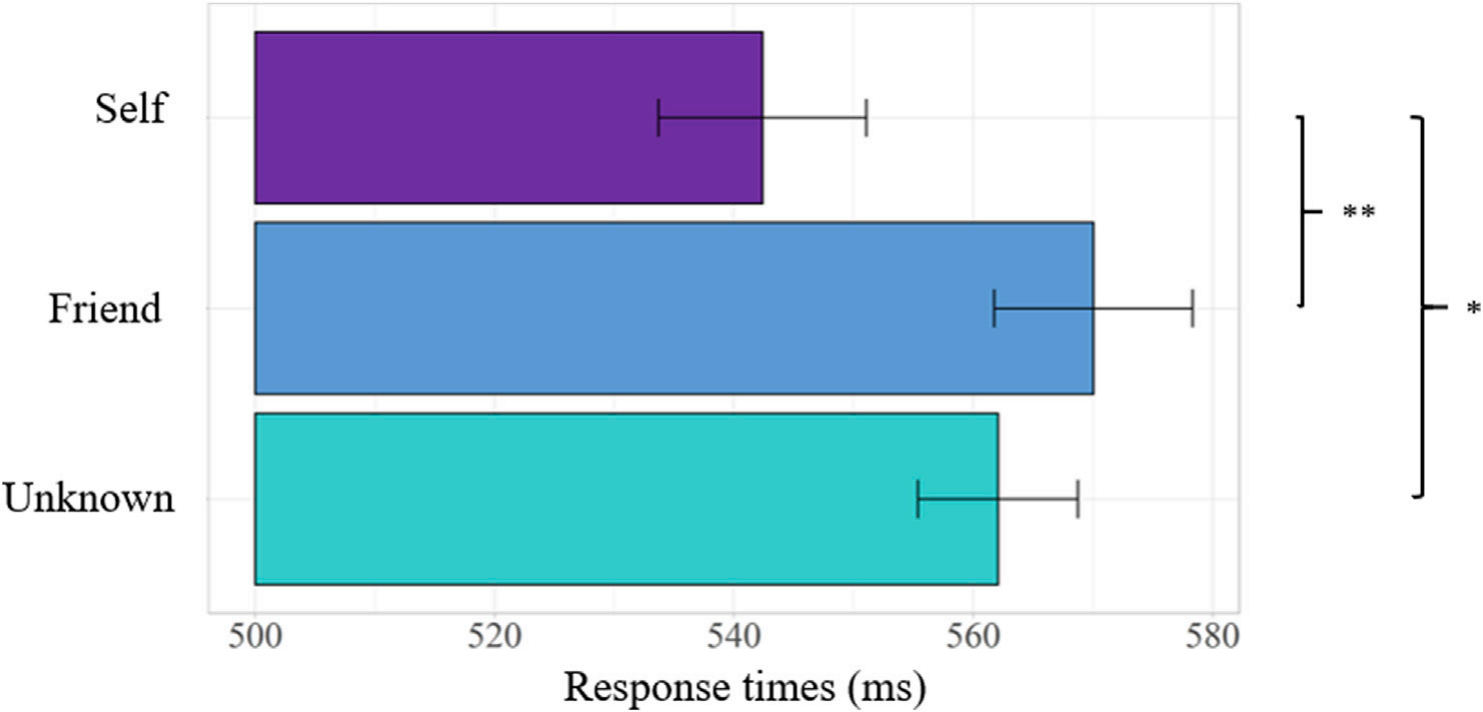
**Mean response times on the facial recognition task.** The figure illustrates the mean response times for the three conditions in milliseconds: *Self*, *Friend,* and *Unknown* faces. Error bars represent Cousineau-Morey confidence intervals ∗p ​< ​.05, ∗∗p ​< ​.01.

Overall, participants achieved a high level of performance on the facial recognition task (mean ​± ​SD hits across all conditions 94.2 ​± ​2.5%). Statistical analysis revealed that the three experimental conditions (*Self, Friend, Unknown*) did not differ in terms of accuracy (F _(2, 48)_ = .796; *p* ​= ​.383, *η*^2^_p_ ​= ​.032). In contrast, a one-way ANOVA revealed a significant effect of face identity on response times (F _(2, 48)_ ​= ​6.861; *p* ​= ​.003, *η*^2^_p_ ​= ​.222). Post-hoc comparisons showed an advantage in self-face recognition (Fig. 2); that is, shorter response times for recognizing the *Self*-face (542 ​± ​10 ​ms) in comparison with either a *Friend* (570 ​± ​10 ​ms; *t*_(24)_ ​= ​−3.172, *p* ​= ​.004, 95% CI [-0.045, -.009], *d* =.614) or an *Unknown* face (562 ​± ​8 ​ms; *t*_(24)_ ​= ​−2.684, *p* ​= ​.013, 95% CI [-0.034, -.004], *d* =.519). These results support the notion that self-face is processed in a distinctive way, and not simply as a familiar face.

### 3.2 EEG results

**Fig. 3:**
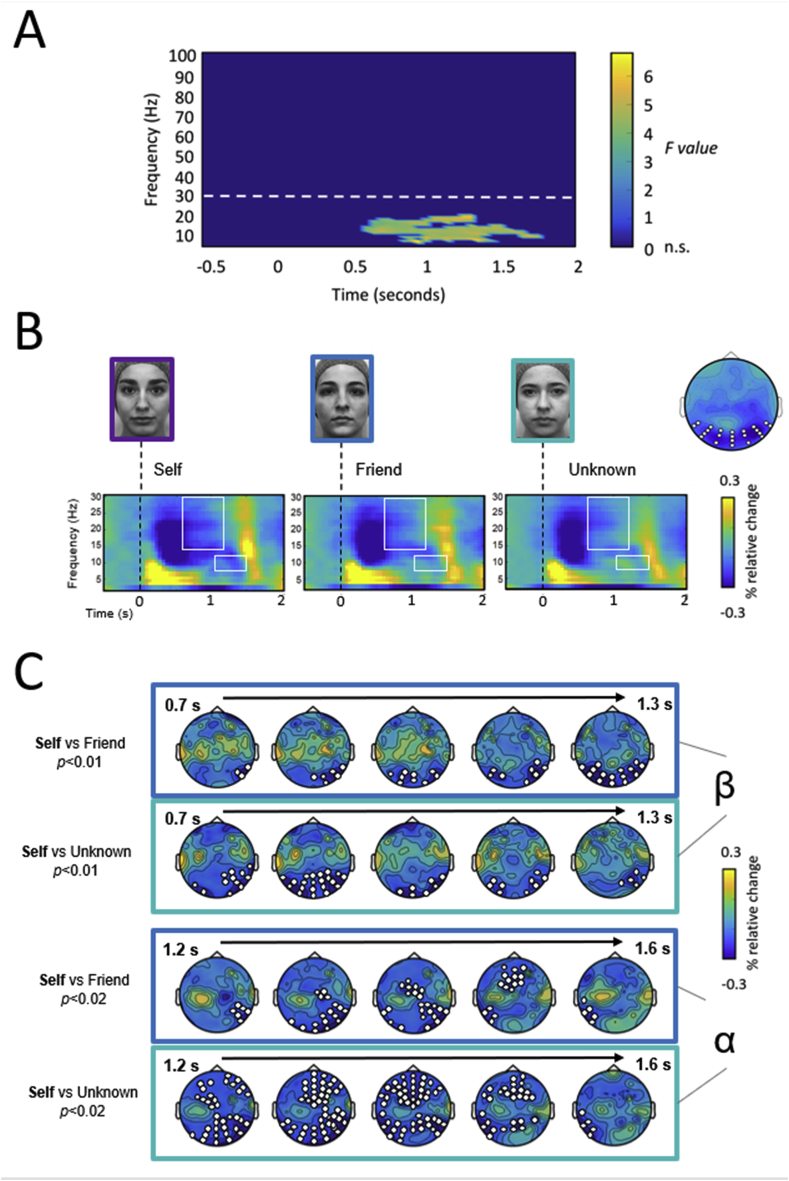
**Top-down attentional modulation during self-face processing.** (A) Time-frequency representation of the F-value collapsed across electrodes for all of the time-frequency ranges (−0.5 to 2 ​s, and 2.5–100 ​Hz). (B) Time-frequency power maps (from 2.5 to 30 ​Hz) of the three experimental conditions (*Self, Friend* and *Unknown* faces). These represent the average time-frequency activity of the group of electrodes showing the largest differences between conditions. The mean topography of all differences between conditions in the alpha-beta range is shown on the right side; selected electrodes employed to compute time-frequency maps are highlighted in white. Alpha and beta frequency bands exhibited a power suppression that was greater and more sustained after self-face presentation. White squares indicate time-frequency windows for significative clusters found in the observed data (beta: 0.7–1.3 ​s at 13–30 ​Hz; alpha: 1.2–1.6 ​s at 8–13 ​Hz). (C) Topographies of power differences in the alpha and beta bands between conditions. Electrodes for each cluster are highlighted in white.

Having demonstrated a self-bias at the behavioural level, we then carried out a time-frequency analysis of the EEG signal to identify self-specific effects at the neural level. The cluster-based permutation F-test revealed an effect among experimental conditions – *Self, Friend,* and *Unknown* (*p* ​= ​0.004). This corresponded to a cluster in the observed data around the alpha (8–13 ​Hz) and beta (13–30 ​Hz) range (see Fig. 3A). Contrary to our expectations, we did not observe any significant effect in the gamma band.

Fig. 3B shows the time-frequency map of each experimental condition. It is clear that all conditions exhibited a power suppression in the alpha and beta bands, although this was more pronounced and sustained for the self-face. An earlier power increase in theta can also be observed, although we have not focused on this frequency band as it did not statistically differ between conditions. Subsequent cluster-based t-tests for alpha and beta band power confirmed an effect of the *Self* condition in comparison to either a *Friend* or an *Unknown* face (see Fig. 3B). Specifically, for the beta band, the non-parametric cluster-based permutation test revealed a significant cluster extending from 0.7 to 1.3 ​s after stimulus presentation. As can be observed in Fig. 3B, beta power suppression was higher for the *Self* face in comparison with both *Friend* (*p* ​= ​.006) and *Unknown* face (*p* ​= ​.001). Similarly, for the alpha band, the non-parametric cluster-based permutation test revealed a significant cluster which extended from 1.2 to 1.6 ​s. In this case, the *Self* face also elicited a stronger power modulation than both the *Friend* (*p* ​= ​.018) and an *Unknown* face (*p* ​= ​.023). It is important to note that we did not found any significant cluster in the alpha-beta power between *Friend* and *Unknown* faces in any case (*p* ​> ​.3). Finally, as shown in Fig. 3C, statistical effects between *Self* and other faces were observed over occipital and frontocentral sensors.

Overall, our time-frequency results show that the self-face induces a greater suppression of alpha-beta power in comparison with other faces, even those that are familiar. Critically, in the case of the alpha rhythm, power modulation persisted at longer latencies when the facial stimulus was no longer present.

**Fig. 4:**
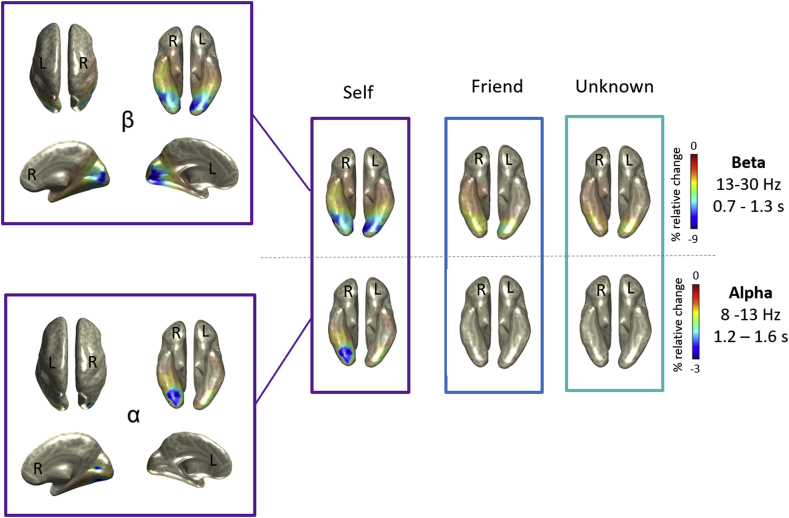
**Brain regions underlying the attentional modulation induced by the self-face.** The figure shows the neural generators of beta (top) and alpha band (bottom) power suppression for the three experimental conditions (*Self, Friend* and *Unknown* faces) during time ranges of interest (0.7–1.3 ​s for beta, and 1.2–1.6 ​s for alpha). The beta source was more broadly distributed over the entire visual cortex, whilst the alpha band was generated around face-related areas in the right hemisphere.

We then carried out beamforming analysis to identify the neural generators underlying beta and alpha power suppression. It is important to bear in mind, however, that we did not have access to individual MRIs. Thus, given that we used the standard MNI template to conduct source localization analysis, our results are only approximate solutions with regard to the locations of brain activity. We found that the alpha rhythm was generated around the intersection between the posterior fusiform gyrus and the inferior/middle occipital gyri, and was strongly lateralized to the right hemisphere. The beta source was more broadly distributed over the occipito-temporal cortex, from primary visual to face-related areas, spanning both hemispheres (Fig. 4). Consistent with the time-frequency analysis, the power decrease of both beta and alpha frequency bands was more pronounced when processing the *Self*-face in contrast to any other face (see Fig. 3).

**Fig. 5:**
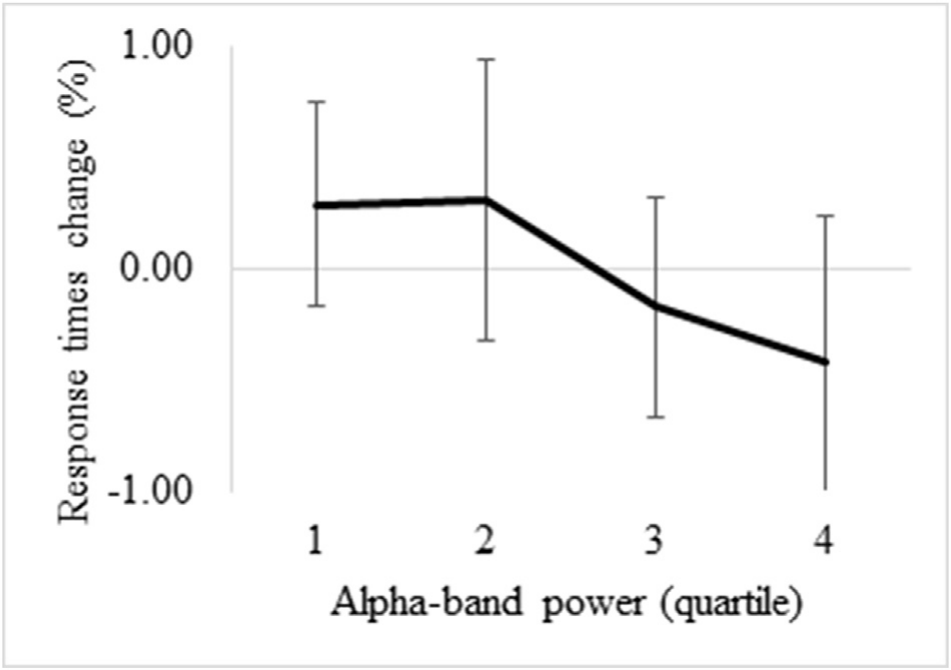
**Relationship between alpha band power in face-related areas and response times in the subsequent trial.** The figure illustrates the percentage change in response times for the different levels of alpha power in the time range between 1.2 and 1.6 ​s. Magnitude of alpha band power is represented in quartile bins, from lowest (1) to highest (4).

We finally speculated whether the alpha band modulation found around face-sensitive brain regions has an influence on processing upcoming facial stimuli. We thus conducted a single-trial analysis to test for a possible relationship between alpha power and behavioural performance. The results showed a slightly negative linear relationship, although it did not reach significance (*r* ​= ​- .104; *p* ​= ​.23; see Fig. 5).

## 4 Discussion

This study aimed to investigate the oscillatory mechanisms of self-face recognition. Time-frequency analysis revealed a greater and sustained decrease in alpha-beta power during self-face processing in comparison with other faces, either familiar or unknown. Critically, alpha band desynchronization was generated in the right occipito-temporal cortex, in the vicinity of brain areas specialized in face processing. Taken together, our results suggest that perceiving one’s own face could trigger a particular attentional mechanism that modulates the activity of cortical regions dedicated to facial perception. Importantly, this effect is specific to the self-face and cannot be explained in terms of familiarity.

As is generally known, sensory stimulation enhances cortical excitability as indexed by high frequency oscillations, while low frequency bands are suppressed (Pfurtscheller & Lopes da Silva, 1999). Here, we did not find evidence for a gamma band power modulation during self-face processing. Nonetheless, it is possible that we have not been able to detect it, since amplitude modulations at high frequencies are subtle and thus may have been overlooked in a statistical analysis based on clusters. In contrast, we did find lower frequency power modulations in the alpha and beta bands, which might reflect the neural implementation of a top-down attentional control mechanism, as we will discuss in the following paragraphs.

Several studies have shown that alpha power decreases over parieto-occipital areas during the deployment and maintenance of attention (Gould et al., 2011; Siegel et al., 2008; Foxe et al., 1998). In particular, when visual attention is focused on a given visual hemifield, alpha power diminishes over the contralateral hemisphere (Ikkai et al., 2016; Kelly et al., 2009; Sauseng et al., 2005). Since alpha suppression is already triggered by the spatial cue that indicates the location of the next stimulus, alpha oscillations have been interpreted as a neural mechanism of top-down attentional control that prepares visual areas for processing upcoming stimuli (Capilla et al., 2014; Gould et al., 2011; Sauseng et al., 2005; Thut et al., 2006). Importantly, we found that alpha band modulation arises from the right occipito-temporal cortex, including the posterior fusiform and the middle and inferior occipital gyri, which are key brain areas in facial perception and recognition (Hasson et al., 2003; Kanwisher et al., 1997; Pitcher et al., 2011; Rossion et al., 2003). This implies that attentional gating does not take place in early visual regions, but rather in downstream areas along the visual ventral pathway, which is in agreement with the findings of previous research (Capilla et al., 2014). Our findings therefore suggest a general role for the alpha rhythm in maintaining relevant brain areas in an “active mode”.

Conversely, the functional role of beta oscillations is less well understood. Beta has been related to the tendency of the sensorimotor system to maintain the ‘status quo’ (Engel and Fries, 2010), although the results of recent research points to its involvement in several cognitive processes including memory retrieval (Hanslmayr et al., 2016) or visual perception (Kloosterman et al., 2015), among others. During sensory stimulation, the decrease in alpha-beta band activity has been related to information processing in specialized cortical modules (Hanslmayr et al., 2016; Jensen and Mazaheri, 2010). In general, the beta rhythm has been associated with the facilitation of endogenous top-down processing (Fries, 2015; Spitzer and Haegens, 2017), and more specifically, it has been proposed to mediate between bottom-up and top-down interactions in the visual cortex (Richter et al., 2018). This role is consistent with the widespread distribution of the beta band source found here, which spanned from early visual to higher-order areas, including face-sensitive regions.

To the best of our knowledge, only one previous study has investigated the brain oscillatory activity induced by one’s own face. Sakihara, Gunji, Furushima and Inagaki (2012) made use of faces (self, familiar, and unknown) and objects to study the mechanisms underlying generic face recognition. They reported an increase in beta band power at 0.4–0.8 ​s over right prefrontal areas during self-face processing. In this case, differences in beta band power were associated with the attentional processes involved in the access to self-related information stored in memory.

In a previous study, we found evidence to suggest that self-face processing is characterized by a reduced need for attentional resources at an early stage (around 200 ​ms), which might facilitate subsequent access to the self-face representation in memory, and therefore recognition (Alzueta et al., 2019). The present study completes this view, by showing a more pronounced alpha-beta power decrease for one’s own face at a later stage, once the face has been recognized. Importantly, alpha band suppression was maintained for a long time even when the face was no longer present, suggesting that an attentional process was being deployed. According to the current Neural Model of the Self (Humphreys and Sui, 2016; Sui and Gu, 2017) self-face processing is built on the interaction between bottom-up and top-down attentional control. Thus, we propose that self-face processing might be driven by bottom-up mechanisms at early stages (Alzueta et al., 2019). Then, once we have recognized our own face by activating its memory representation, top-down control mechanisms would come into play by allocating greater and sustained attentional resources to keep self-face representation in an active state. It is important to note, however, that we did not directly manipulate attention, and therefore our findings are open to alternative interpretations that do not involve attentional processes.

Our suggestion that the self-face effect is most likely explained by a top-down attentional mechanism is reinforced by the source localization results. These results indicate that long-lasting alpha band suppression arises from face-sensitive areas, including fusiform as well as the inferior and middle occipital cortex in the right hemisphere (Dricot et al., 2008; Rossion et al., 2003). Interestingly, previous research has demonstrated that the fusiform gyrus can be modulated by top-down attentional mechanisms (Wojciulik et al., 1998). In line with the present results, neuroimaging studies have shown stronger activation of the right fusiform and inferior and middle occipital gyri during self-face processing in comparison with other faces (see Hu et al., 2016 for a recent review). These areas are particularly engaged during self-face processing (Devue and Brédart, 2011), leading to a reduction in self-prioritization when such areas are lesioned (i.e. a hypo-self-bias) (Sui et al., 2015).

A question that remains open is whether the potential ability of the self to hijack attentional resources might either facilitate or hamper forthcoming stimulus processing. Our data do not provide conclusive evidence to clarify this issue, likely because the experimental task was too easy (with accuracy of around 95%). A more demanding task (e.g. with shorter exposure time to the faces) would be more suitable for testing this hypothesis. Nevertheless, taking into account the accumulated evidence showing that alpha-beta activity enhances sensory processing precision (Bauer et al., 2014; Cravo et al., 2011), and the localization of these effects over face-related regions, we hypothesize that alpha-beta modulation might have a facilitatory role by preparing specialized sensory areas for potentially relevant incoming stimuli.

In addition to one’s own face, other self-related and relevant stimuli, such as the self-name, also mobilize more attentional resources (Tacikowski and Nowicka, 2010). Hence, the greater deployment of attention for the self may be an adaptive neural mechanism to facilitate subsequent processing of socially relevant information. If this were the case, seeing one’s own face would command attention in a similar way to hearing one’s own name. This argument has received support from a recent ERP study (Woźniak et al., 2018), which demonstrated that the presentation of self-related stimulation facilitated subsequent processing of any other stimulus, whether associated with the self or with other identities. The facilitatory effect of the self has also been evidenced by a recent study that employed faces as spatial cues in a dot-probe task (Wójcik et al., 2018). These authors observed that participants were faster at detecting the target when it was preceded by the self-face in comparison with targets preceded by other faces.

Moreover, the interesting eye-tracker study carried out by Devue et al. (2009) is worth noting here. These authors presented the participant’s self-face, among others, during a visual search task. In agreement with the results obtained in the present study, they found that once fixation to the self-face had been established it was difficult to disengage attention from it. However, in contrast with our results, they also found a similar effect when the faces were those of friends. We believe that this conflicting evidence, which can also be found in other studies (Alexopoulos and Muller, 2012; Brédart and Devue, 2006; Devue and Brédart, 2008; Keyes and Dlugokencka, 2014), might be explained by a possible participant identification with the stimuli. According to the expanding nature of the self (Aron and Fraley, 1999; Mattan et al., 2016; Sui et al., 2013; Sui and Humphreys, 2013), it could be hypothesized that a familiar face (e.g. a partner’s face) can sometimes be processed as a familiar face, but also as a self-related stimulus as if it were part of the self (see Taylor et al., 2009). It is therefore possible that personal identification with the stimuli, as well as the experimental tasks employed in different studies, could have turned familiar stimuli into self-related stimuli, thus accounting for the contradictory results found in the literature. This is a very interesting question that deserves further investigation.

Finally, it could be argued that the self-face is simply an extremely familiar face, and therefore our findings could ultimately be the result of a familiarity effect. However, in a recent ERP study, Alzueta et al. (2019) found support for the view that the self-face is a distinctive face stimulus, since it was processed differently to both familiar and unknown faces at an early stage (i.e., P200 component). In contrast, familiar and unknown faces did not differ at this stage, but did so later at the N250 latency, when familiarity is computed. The strong evidence that supports this notion comes from a study by Woźniak et al. (2018), who found that brain activity at early latencies are specific to the self and cannot be attributed to familiarity. Specifically, they employed a task in which participants had to associate newly learned faces with either themselves, a friend, or a stranger. They also found a reduced amplitude at anterior electrodes in response to the self-face at around 200 ​ms, which could not be explained by familiarity with the self-face, since all faces were equally unknown to the participants at the beginning of the experiment. Our present results also support the view that the self-face is processed as a distinctive face stimulus rather than a highly familiar face, given that: (i) alpha-beta power differed for the self-face in comparison with both familiar and unknown faces, and (ii) we did not find any statistical difference between the friend and unknown faces. Since this pattern of results is highly reminiscent of the P200 effect, which Woźniak et al. (2018) found to be specific to the self, we suggest that the oscillatory effects found in the present study are most likely explained by a real self-face effect, rather than by familiarity.

In conclusion, our results show a greater and more sustained reduction in alpha-beta band power during self-face processing in face-sensitive brain regions in comparison with other faces, suggesting the operation of a specific top-down attentional mechanism. As in the Myth of Narcissus, who became caught by his own reflection, our results suggest that one’s own face is able to retain attention, which has led us to refer to this phenomenon as the ‘Narcissus Effect’. We propose that this mechanism might play an adaptive role by facilitating the processing of subsequent personally relevant information. Our findings might also have important implications for neuropsychiatric research, since the self and its attentional mechanisms are altered in certain mental disorders.

## CRediT authorship contribution statement

**Elisabet Alzueta:** Conceptualization, Methodology, Formal analysis, Investigation, Visualization, Writing - original draft. **María Melcón:** Formal analysis, Writing - review & editing. **Ole Jensen:** Methodology, Writing - review & editing. **Almudena Capilla:** Supervision, Funding acquisition, Methodology, Software, Writing - review & editing.
